# Current postgraduate training in emergency medicine in the Nordic countries

**DOI:** 10.1186/s12909-023-04430-x

**Published:** 2023-06-23

**Authors:** Hjalti Már Björnsson, Lars Petter Bjørnsen, Christian Baaner Skjærbæk, Katrin Hruska, Ari Palomäki, Nordic EM Study Group, Tommy Andersson, Christian Rasmussen, Ulf Grue Hørlyk, Ville Hällberg, Teemu Koivistoinen, Jonni Unga, Bahram Shams, Cornelia Härtel

**Affiliations:** 1 Faculty of Medicine, University of Iceland, Reykjavík, Iceland; 2 Department of Emergency Medicine, Landspitali-The National University Hospital of Iceland, Fossvogur, 108 Reykjavík, Iceland; 3 St. Olav’s University Hospital, St. Olav’s University Hospital, Trondheim, Norway; 4 Department of Circulation and Medical Imaging, Norwegian University of Science and Technology, Trondheim, Norway; 5 Emergency Department, Regionshospitalet Randers, Randers, Denmark; 6 Capio Rapid Response Vehicles, Stockholm, Sweden; 7 Faculty of Medicine and Health Technology, Tampere University, Tampere, Finland; 8 Emergency Department, Kanta-Häme Central Hospital, Hämeenlinna, Finland

**Keywords:** Post-graduate medical education, Nordic countries, Emergency medicine

## Abstract

**Background:**

Emergency Medicine (EM) is an independent specialty in all five Nordic countries. This study aims to evaluate the structure of post-graduate EM training in the area.

**Methods:**

A leading hospital or hospitals in EM training in each country were identified. An e-survey was sent to each hospital to gather data on patient volume and physician staffing, curriculum, trainee supervision, and monitoring of progression in training.

**Results:**

Data were collected from one center in Iceland and Norway, two in Finland and Sweden, and four centers in Denmark. The data from each country in Denmark, Finland, and Sweden, were pooled to represent that country. The percentage of consultants with EM specialist recognition ranged from 49–100% of all consultants working in the participating departments. The number of patients seen annually per each full time EM consultant was almost three times higher in Finland than in Sweden. In Iceland, Denmark, and Sweden a consultant was present 24/7 in the ED but not in all centers in the other countries. The level of trainee autonomy in clinical practice varied between countries. Requirements for completing standardized courses, completing final exams, scientific and quality improvement projects, and evaluation of trainee progression, varied between the countries.

**Conclusions:**

All Nordic countries have established EM training programs. Despite cultural similarities, there are significant differences in how the EM training is structured between the countries. Writing and implementing a standardized training curriculum and assessment system for EM training in the Nordic countries should be considered.

**Supplementary Information:**

The online version contains supplementary material available at 10.1186/s12909-023-04430-x.

## Background

The model of emergency medicine (EM) is based on having fully trained emergency physicians (EP) providing the initial assessment and care for all patients presenting acutely to the emergency department (ED). Since this model was first introduced in the United States over half a century ago, it has become the dominant model of providing emergency care in the world [1–4].

Over the last quarter century there has been a slow but gradual development of EM in the Nordic countries. The first country to fully recognize EM as an independent specialty was Iceland in 1992, followed by Finland in 2013, Sweden in 2015, Denmark in 2018 and finally Norway in 2019 [5–7]. Adopting and fully integrating the EM model of care does not however happen overnight. In all countries that have recognized EM as an independent specialty, it has been a process that takes several years or decades to fully incorporate the model of EM into the healthcare system.

All Nordic countries currently have postgraduate training programs for specialist training in EM but are at various stages in developing a full EM training program. Despite cultural similarities between the Nordic countries, and a similar structure of their health care systems, there generally has not been much collaboration between them with regards to postgraduate medical education. In critical care and palliative medicine, a combined Nordic training program for each respective specialty has been successfully implemented but the general rule has been that each Nordic country has set up its own curriculum and training pathway for each medical specialty [8, 9].

As EM is a relatively new specialty, it might be feasible to design and implement a combined Nordic EM training curriculum. This would both facilitate creating dedicated teaching material and assessment system, as well as open options for trainees to rotate between the different countries for specific components of their training. A first step towards such a combined Nordic EM curriculum is to assess the current approach to postgraduate EM training in each of the Nordic countries.

The aim of this study is to gather comparative data on how EM training is currently done in the five Nordic countries.

## Methods

In each country, a leading hospital or hospitals in EM training were identified by an investigator from each country. An electronic survey was sent to the training program directors (TPD) to collect data on each training center.

The liaison for each hospital was requested to provide the number of EM consultants and trainees at their institution and annual number of patients seen in their department. They were also requested to provide information on clinical supervision of trainees, lecturing and hands on courses, documentation of training delivered, requirements of trainee involvement in research and quality improvement projects and requirements for progression in training and how training is completed. Finally, information on how government and other external credentialing of training sites is handled was also requested. Data were collected and managed using REDCap electronic data capture tools hosted at the University of Iceland [10, 11].

## Results

Data was entered from a total of 10 EM training centers. In Iceland and Norway, one center was considered to be spearheading EM development. In Finland and Sweden, two centers were included from each country. In Denmark it was considered impossible to identify definitive leading centers, so data was gathered from four centers considered to represent the general status of EM training programs in the country. When analyzing the data from countries with more than one participating training center, the data from each country as pooled to represent that country.

Postgraduate EM training was a 6-year program in Iceland and Denmark but a 5 year program in the other three countries. In all countries, completing a period of foundation training is required after medical school, ranging from 1–1,5 years.

The annual Emergency Department (ED) patient volume ranged from 27,000 to 110,000. The number of trainees ranged from 2 to 44 in each participating department and the number of consultants ranged from 4 to 36. The percentage of consultants with specialist recognition in EM ranged from 49 to 100%. When looking at the number of patients with regards to physician staffing combined for each country, the number of patients seen in the ED every year was divided by the number of Full Time Equivalent (FTE) consultants working clinically in the departments. In Sweden there were 3063 patients seen in the EDs per FTE consultant whereas in Finland this number was almost three times higher or 8010 patients/consultant FTE/year (Table 1).

**Table 1:**
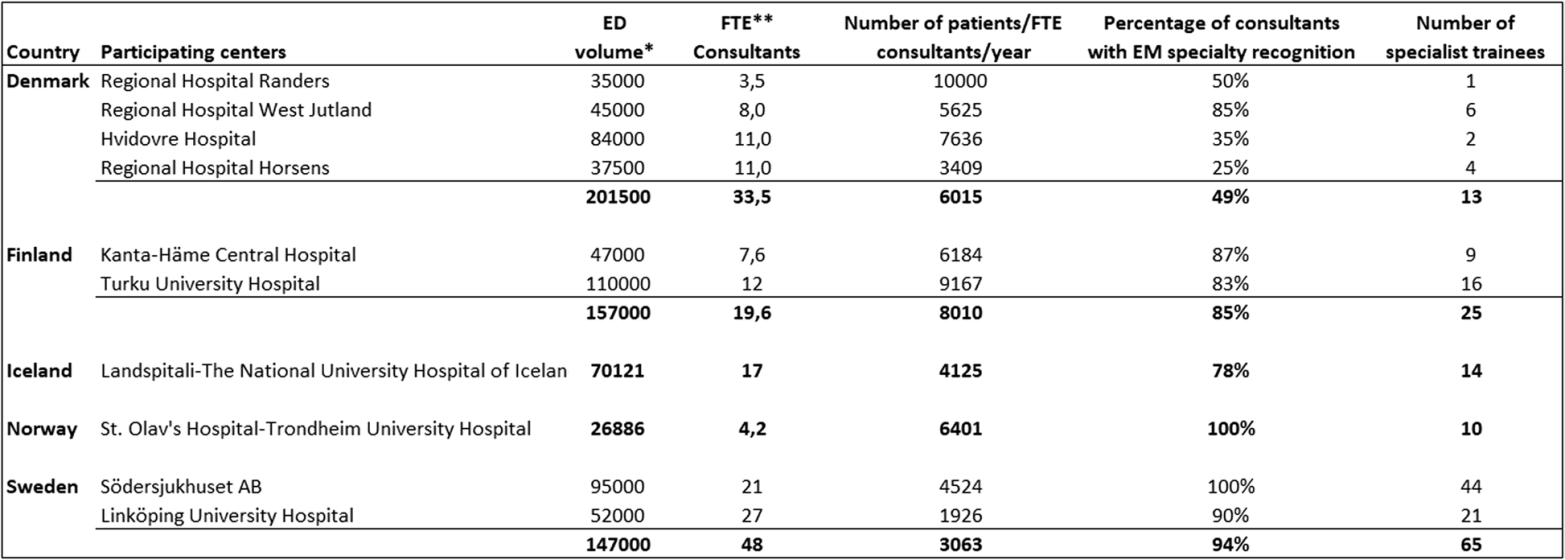
Number of consultants, trainees, and patients

^*^Annual number of patients seen in the Emergency Department

^**^Full time equivalent

### Trainee clinical supervision

In Denmark, Iceland, and Sweden a consultant was physically present 24/7 in the ED but in Norway and one of the departments in Finland, trainees were working without direct consultant supervision during overnight shifts.

For foundation trainees, in all centers in Norway, Iceland, and Sweden, Iceland and in three of the four centers in Denmark, the trainees were required to discuss all patients with a consultant who also sees the patients independently if needed. In Finland, foundation trainees were given more autonomy and only expected to discuss cases in major care with a consultant but could independently manage patients in minor care.

All centers allowed for gradually increased trainee autonomy as the training progressed. At the third or fourth year, all but one center allowed trainees to practice independently except that a consultant was always present for major cases such as cardiac arrest, major trauma, or airway management. By the final year of training, all but Norway allowed trainees to practice independently with a consultant being available only when the trainee felt he or she needed consultant backup. The detailed listing of trainee supervision is presented in Table 2.

**Table 2:** Trainee clinical supervision

	**Consultant presence in the ED, average hours/day**	**Average level of consultant clinical supervision of trainee**^a^						
		**Foundation training-Internship (AT)**	**1st year**	**2nd year year**	**3rd year**	**4th year**	**5th year**	**6th year**
**Denmark**	24	2,0	2,5	2,8	3,5	4,0	5,0	4,7
**Finland**	19	3,5	4,0	4,5	4,5	5,0	5,5	5,5
**Iceland**	24	2,0	2,0	2,0	3,0	5,0	5,0	6,0
**Norway**	13	1,0	2,0	3,0	3,0	4,0	4,0	
**Sweden**	24	1,5	2,0	2,5	3,5	4,5	5,0	5,5

^a^

1Trainee discusses all patients with a consultant who independently also sees all patients

2Trainee discusses all patients with a consultant who also sees the patients independently if needed

3Trainee discusses all patients in major care with a consultant but does not discuss all patients in minor care with a consultant

4Trainee practices independently except that a consultant is always present for major cases, cardiac arrest, major trauma or airway management

5Consultant is available in the ED but only supervises the trainee if trainee feels needed

6Consultant is available on call if trainee feels needed but not 24/7 physically present in the ED

### Training program organization

In Denmark, Finland, Norway, and Sweden a national curriculum had been written and was used for the training program, in Iceland a curriculum from the UK had been adopted for national use. All programs had some form of credentialing of the EM training program. This was done by a national external committee in all centers. Iceland was the only center that reported credentialing by both a national and an international committee.

All training programs had a dedicated training program director (TPD). The amount of protected time for this task was very variable, ranging from 5 to 100% with most centers reporting 40–50% protected teaching time for the TPD. In Iceland, Norway, and Sweden, all centers had a training faculty group overseeing the program, this was present in one of the two centers in Finland and in one of the four centers in Denmark. In all but one program in Finland, trainees had a designated Educational Supervisor (ES) during their training and were required to meet with their ES every one to four months. The ES had received training for this role in Iceland, Norway, Sweden, and in Denmark but in neither participating center in Finland.

Clinical teaching was documented in all programs except for one in Sweden and one in Finland. All other reported documenting Directly Observed Procedures (DOPs). Direct observations of clinical history taking and physical exam by trainee and Case Based Discussions (CBD) were documented in Iceland, Norway, and half of the centers in Denmark, Finland, and Sweden. All programs reported formal training with 2–6 h per week of lectures and 0.5–6 h of simulation and hands-on training.

### Standardized training courses

All but one center in Sweden required trainees to complete Advanced Cardiac Life Support (ACLS) or equivalent training and all but one center in Finland required an Advanced Trauma Life Support (ATLS) or equivalent course. Completing a Pediatric Advanced Life Support (PALS) course or equivalent was required in Denmark, Iceland, and Norway and in one center in Sweden but not required in Finland. Iceland was the only center that required trainees to complete a difficult airway course (Table 3).

**Table 3:** Required standardized courses

Country	Advanced Cardiac Life Support	Advanced Pediatric Life Support	Advanced Trauma Life Support	Difficult airway
**Denmark**	Required in all programs	Required	Required	Not required
**Finland**	Required in all programs	Not required	Required in ½ programs	Not required
**Iceland**	Required	Required	Required	Required
**Norway**	Required	Required	Required	Not required
**Sweden**	Required in ½ programs	Required in ½ programs	Required	Not required

ACLS- Advanced Cardiac Life Support

APLS- Advanced Pediatric Life Support

ATLS- Advanced Trauma Life Support

### Annual progression of trainees

The progression of all trainees in Iceland and Norway and in one center in Finland was formally evaluated by an external panel every year.

In one of the centers in Finland and all centers in Denmark and Sweden, annual progression of training was evaluated by the Educational Supervisor.

### Final exams

In Finland and Iceland, all trainees were required to pass a board exam to obtain a specialist license. In Iceland, both a formal knowledge exam and a clinical exam were required, while in Finland, only a written knowledge exam was obligatory, and a clinical skills exam was optional. In Sweden both exams were optional for the trainees. In Norway and Denmark, no exams were required.

### Scientific research

In all centers in Sweden and Denmark, trainees were required to complete a research project. In Iceland, Norway, and one of the centers in Finland, scientific research was optional for those interested in academic research. One center in Finland reported no research training provided within the EM training program.

### Quality Improvement Projects

All trainees in Iceland, Norway, and Sweden, and in one center in Denmark are required to complete a Quality Improvement Project (QIP). Such projects are optional in other centers.

## Discussion

Training programs in EM have been established in all five Nordic countries. Although the health care systems are quite similar in all countries, this study illustrates that there are significant differences in how the EM training programs are structured.

In most centers, most or all ED consultants had a specialist certification in EM. Denmark appeared to still be in the early stages of establishing the specialty as just less than half of the ED consultants were reported to have an EM specialist certification. In the centers that were still lacking fully trained EM specialists to teach the EM trainees, the ED was often staffed with consultants that had completed training in other specialties. It is important that these consultants receive additional training for this role as has been successfully done in other countries adopting EM [1, 5].

In the leading EM centers in each country, trainees appeared to be mostly supported in their work by direct consultant supervision. This is essential to ensure the quality of both the training and care provided. The training centers generally allowed the trainee to gradually take more responsibility with progression in training.

Finland appeared to be an outlier in this regard as foundation level and specialist trainees in the first two years were allowed much more independent practice than was done in the other countries. This seems to be largely due to lack of EM consultants, as the number of patients treated in the ED every year per each consultant in Finland was two to three times the number treated per consultant per year in the other countries. However, a new EM training program was launched in Finland in late 2020 where trainees are closely supervised with the help of CBD, DOPS and direct observation of history taking and physical exam. As the number of fully trained EM consultants is also increasing in Finland, it is expected that training will rapidly progress in the next few years.

All Nordic countries have chosen to write or adopt their individual curriculum for EM training for national use. There were also significant discrepancies in how trainees were supervised, mentored, clinical teaching documented, trainee progression monitored and how training was completed. As the health care systems are generally quite similar in terms of undergraduate training, roles of different health care professionals, funding, and patient population, it was surprising to see that there did not seem to be much collaboration among Nordic emergency physicians with regards to structuring post graduate training.

Completing standardized courses in the approach to specific acute presentations, such as the ACLS, ATLS, and PALS, is generally considered beneficial for EM trainees in countries with a longer history of EM training. Denmark and Iceland were the only countries where completing all of these standard courses was required. Incorporating these courses into the EM training in all countries could be a helpful step in advancing EM care in the Nordic countries as many other specialties consider them essential for training physicians to provide adequate emergency care. This is however debated as The American College of Emergency Physicians (ACEP) has issued a statement objecting the requiring of such short courses a requirement for EM practice and feels that what the training provided in these courses are a part of the core curriculum in EM [12]. It is considered especially redundant to require fully trained actively practicing consultants to repeat those courses at regular intervals when they routinely perform the acute interventions in their regular work.

Emergency physicians need to be well trained to perform any acute lifesaving intervention that a patient may need immediately. Being able to manage the airway certainly is one of these acute interventions and is a core competency for any emergency physician. In the Nordic countries, anesthesiologists have traditionally managed the airway and in many centers been reluctant to acknowledge the skill and expertise of a fully trained EM specialist in this role. This is in spite of the fact that numerous studies have shown that emergency physicians have similar outcomes in emergency airway management in the ED as anesthesiologists even if they perform intubations less frequently [13–17]. Iceland is the only center that required completing a specific course on difficult airway management. This has been taught in collaboration between the departments of anesthesia and EM and has been very helpful in increasing mutual respect and collaboration between the two groups of consultants. Adopting such a course into the training in other countries could be helpful in advancing airway management by Nordic EM physicians.

Scientific research is the foundation on which all medical care is based. In most areas where EM training is well established, completing a research project is not required to become a fully credentialled EM physician. In the Nordic countries, the Danish and Swedish EM trainees were required to complete a research project but in other countries research activities were either optional or not required. Scientific research contributes to the global knowledge in how to provide EM care and the academic progression of EM consultants [18–22]. However, a well-functioning ED can be established without any research activities. The decision on how much emphasis is placed on research activities may depend on ED staffing, funding, and administrative support in the hospital. If too much emphasis is placed on academic research this may negatively affect the clinical training and staffing of the ED.

In the recent years the requirements of trainees to complete a QIP have increased [23–25]. It is now generally considered necessary to train future consultants both in the role to provide good clinical care, and to have the knowledge to assess and improve the functions of their ED. Many trainees find this work rewarding and relatively easy to accommodate with the often-busy life of a post-graduate medical trainee. Completing a successful QIP can also be an important step for the trainee to establish a role within their department in not just seeing patients but also improving patient care. As many of the EDs in the Nordic countries are still in the early stages of adopting the EM model of care, it is important that consultants receive training in improving their department. Trainees in Iceland, Norway, and Sweden, and one center in Denmark were required to complete a QIP project. In Denmark and Finland, this should be considered as an option to advance EM care.

## Limitations

This study only gathered information from specific EDs in each country. This was done in an attempt to obtain data on how training was approached in the most advanced centers, with the assumption that it would represent in what direction other centers in that country were heading with their training program. As there are multiple centers in Denmark, Finland, Norway, and Sweden providing EM training in some form, our results do not necessarily represent the current average approach to EM training in those countries. Data were not collected on how patients were divided between urgent care centers or the ED in different health systems, this may have influenced the number of patients managed by the consultants in the department.

## Conclusions

EM training programs have been established in all Nordic countries but with minimal standardization and collaboration between the countries. Significant differences were found in how the training was structured, trainee supervised, progression monitored and what was required for completing training. Writing and implementing a combined standardized training curriculum and assessment system for EM training in the Nordic countries should be considered.

## Supplementary Information

**Additional file 1.** Survey.

## Data Availability

All data generated or analysed during this study are included in this published article.
