# Supplementary material for: Current postgraduate training in emergency medicine in the Nordic countries
Source: BMC Med Educ. 2023 Jun 23;23:469. doi: 10.1186/s12909-023-04430-x (PMC10288667; doi:10.1186/s12909-023-04430-x)
Supplement: Supplementary file 1 — Additional file 1. Survey. [file 12909_2023_4430_MOESM1_ESM.pdf]

Example Survey

These are your survey instructions that you would enter for your survey participants. You may put whatever text you like here, which may include information about the purpose of the survey, who is taking the survey, or how to take the survey.

Surveys can use a single survey link for all respondents, which can be posted on a webpage or emailed out from your email application of choice. By default, all survey responses are collected anonymously (that is, unless your survey asks for name, email, or other identifying information). If you wish to track individuals who have taken your survey, you may upload a list of email addresses into a Participant List within REDCap, in which you can have REDCap send them an email invitation, which will track if they have taken the survey and when it was taken. This method still collects responses anonymously, but if you wish to identify an individual respondent's answers, you may do so by also providing an Identifier in your Participant List. Of course, in that case you may want to inform your respondents in your survey's instructions that their responses are not being collected anonymously and can thus be traced back to them.

This survey is intended to be filled out by the training program director for the post-graduate emergency medicine training program.

Name of person filling out survey	______________________
Email	______________________
Country	◯ Denmark<br>◯ Finland<br>◯ Iceland<br>◯ Norway<br>◯ Sweden
Name of hospital	______________________
Number of patients seen in the ED every year	______________________
What is the number of consultants working in your ED?	______________________
How many full time equivalent (FTE) consultants are working in your department? If you have a consultant who all does half of his/her workload in teaching or research and half of the time as clinical work in the ED, this consultant counts as 0.5 FTE?	______________________
What percentage of the EM consultants have a specialist recognition in Emergency Medicine?	______________________
Does the program have a designated training program director?	◯ Yes ◯ No
How much protected time does the TPD have to manage the program? Please provide percentage of their workload that is for the program.	______________________

Does the training program have a designated training faculty group? ◯ Yes ◯ No

How many foundation training (AT) trainees are currently working in your department? ______________________

How many specialist trainees (ST) in Emergency Medicine are currently in the EM program at your hospital? Please include all who are in the program, both those that are currently in the ED and those who are on rotations in other departments. ______________________

This section is to document specialist presence in the ED for the different times of the day and week. Please describe the number of hours every day of the week that a specialist is physically present in the ED to supervise trainees.

	1	2	3	4	5	6	7	8	9	10	11	12	13	14	15	16	17	18	19	20	21	22	23	24
Monday	◯	◯	◯	◯	◯	◯	◯	◯	◯	◯	◯	◯	◯	◯	◯	◯	◯	◯	◯	◯	◯	◯	◯	◯
Tuesday	◯	◯	◯	◯	◯	◯	◯	◯	◯	◯	◯	◯	◯	◯	◯	◯	◯	◯	◯	◯	◯	◯	◯	◯
Wednesday	◯	◯	◯	◯	◯	◯	◯	◯	◯	◯	◯	◯	◯	◯	◯	◯	◯	◯	◯	◯	◯	◯	◯	◯
Thursday	◯	◯	◯	◯	◯	◯	◯	◯	◯	◯	◯	◯	◯	◯	◯	◯	◯	◯	◯	◯	◯	◯	◯	◯
Friday	◯	◯	◯	◯	◯	◯	◯	◯	◯	◯	◯	◯	◯	◯	◯	◯	◯	◯	◯	◯	◯	◯	◯	◯
Saturday	◯	◯	◯	◯	◯	◯	◯	◯	◯	◯	◯	◯	◯	◯	◯	◯	◯	◯	◯	◯	◯	◯	◯	◯
Sunday	◯	◯	◯	◯	◯	◯	◯	◯	◯	◯	◯	◯	◯	◯	◯	◯	◯	◯	◯	◯	◯	◯	◯	◯

Comments ______________________

This section is to document the level of supervision of trainees in your emergency department. Please list the general level of supervision provided for a trainee at each level of training according to the listed categories:
1. Trainee discusses all patients with a consultant who independently also sees all patients.
2. Trainee discusses all patients with a consultant who also sees the patients independently if needed.
3. Trainee discusses all patients in major care with a consultant but does not discuss all patients in minor care with a consultant.
4. Trainee practices independently except that a consultant is always present for major cases such as cardiac arrest, major trauma and airway management.
5. Consultant is available in the ED but only supervises the trainee if trainee feels needed.
6. Consultant available on call if trainee feels needed but not 24/7 physically present in the ED.

	1	2	3	4	5	6

Foundation training - Internship (AT)	◯	◯	◯	◯	◯	◯
1st year	◯	◯	◯	◯	◯	◯
2nd year	◯	◯	◯	◯	◯	◯
3rd year	◯	◯	◯	◯	◯	◯
4th year	◯	◯	◯	◯	◯	◯
5th year	◯	◯	◯	◯	◯	◯
6th year	◯	◯	◯	◯	◯	◯

Comments ______________________________

What curriculum is used in the EM training program?
- ◯ National curriculum issued by a national EM society
- ◯ Other curriculum adopted for national use
- ◯ No formal curriculum is used

Is the curriculum used in the EM training program compatible with the EuSEM EM curriculum?
- ◯ Yes
- ◯ No

How is the EM training program credentialed to provide EM training by national authorities?
- ☐ The program has been reviewed by an international external committee and formally credentialed to provide EM training.
- ☐ The program has been reviewed by an external national committee and formally credentialed to provide EM training.
- ☐ The program has been reviewed by a local hospital committee who credentials it to provide EM training.
- ☐ No formal credentialing of the training program has been done.

How many hours per week on average do the trainees get formal teaching time as lectures? ______________________________

How many hours per week on average do the trainees get formal teaching time as simulation and hands on training? ______________________________

Which of the following courses are mandatory for EM trainees to complete?
- ☐ Advanced Cardiac Life Support (ACLS or equivalent)
- ☐ Advanced Trauma Life Support (ATLS or equivalent)
- ☐ Pediatric Advanced Life Support (PALS or equivalent)
- ☐ Difficult Airway Course

What other courses are trainees required to complete? ______________________________

Do trainees have a designated Educational Supervisor (ES) during their training?
- ◯ Yes, all trainees have a designated ES
- ◯ Some trainees have a designated ES
- ◯ No trainees have a designated ES

How often does the trainee meet with the ES as a minimum?	◯ Once per year<br>◯ Twice per year<br>◯ Every 4 months<br>◯ Every 3 months<br>◯ Every other month<br>◯ Every month<br>◯ Not specified
Have the Educational Supervisors received specific training for their role as ES	◯ Yes<br>◯ No
How is the delivered clinical teaching documented? Please select all that apply.	☐ Clinical teaching is not formally documented<br>☐ Requirements of documented direct observations of clinical history taking and physical exam by trainee<br>☐ Requirements of documented case based discussions with trainee<br>☐ Requirements of documented directly observed procedures performed by trainee.
How much scientific research training is provided within the EM training program?	◯ None<br>◯ Scientific research is optional for those interested in academic research<br>◯ All trainees are required to complete a research project
Ho much training in Quality Improvement management is provided within the EM training program?	◯ None<br>◯ Optional for those interested in QI projects<br>◯ All trainees are required to complete a QI project
How is the annual progression of a trainee assessed in the program?	◯ No formal process<br>◯ Formal annual evaluation by Educational Supervisor<br>◯ Formal annual evaluation by an external panel
Are trainees required to pass a formal knowledge exam to obtain a specialist recognition?	◯ Yes<br>◯ Taking an exam is optional<br>◯ No exam exists
Are trainees required to pass a formal clinical exam to obtain a specialist recognition?	◯ Yes<br>◯ Taking an exam is optional<br>◯ No exam exists
